# Evaluation of Psychometric Properties of the Farsi Version of the Work-Related Affective Feelings Scale

**DOI:** 10.11621/pir.2025.0204

**Published:** 2025-06-01

**Authors:** Roghieh Nooripour, Nahid Hoseininezhad, Parviz Fadakar Gabalou, Davod Fathi

**Affiliations:** a Islamic Azad University, Qazvin, Iran; b Alzahra University, Tehran, Iran; c Allameh Tabataba’i University, Tehran, Iran; d University of Mohaghegh Ardabili, Iran

**Keywords:** work-related affective feelings, psychometric properties, employees’ emotions, personality

## Abstract

**Background:**

Attention to emotions is growing in workplaces. To address this interest, new psychological instruments have been developed to assess the emotional states of individuals at work. The Work-Related Affective Feelings (WORAF) Scale is a new psychological tool for measuring four main emotions: happiness, anxiety, anger, and dejection at work.

**Objective:**

To determine psychometric properties of the Farsi version of the WORAF Scale in Iran.

**Design:**

This research employed a cross-sectional research design. The sample consisted of 514 people (250 females and 264 males). We collected data using the Work-Related Affective Feelings Scale, the Job-Related Affective Well-Being Scale, and the Ten-Item Personality Inventory. Confirmatory Factor Analysis was used to check the factorial structure of this scale. The Pearson correlation coefficient and Cronbach’s alpha were analyzed using SPSS version 26 and R software version 4.2.

**Results:**

The model fit indices suggested satisfactory fit for the final models as CFI = .907, TLI = .904, NFI = .907, IFI = .907, X^2^/_df_ = 3.809, RMR = .055, and RMSEA = .079. The Cronbach’s alpha of the scale was .87 and values ranging from .73 to .92 confirmed the reliability of the WORAF. The Pearson correlation analysis revealed a statistically significant relationship between this scale and similar scales, verifying the convergent validity of this scale and similar scales.

**Conclusion:**

The findings provide empirical support and introduce the WORAF Scale as a valid and reliable scale translated and validated in Iran. The Farsi version of this scale can measure four major emotions of individuals in Iranian workplaces. This scale can be used by Iranian researchers as well as by organizational practitioners to measure, identify, and improve the emotions employees experience in their workplaces. Considering the large number of employees in the private and public sectors in Iran, this scale can be useful.

## Introduction

Understanding the roles of employees’ emotions is critical for effective human resource management in contemporary organizations, as most individuals spend over 50% of their lives at work (Xerri et al., 2023). The occupational setting and the specific nature of an employee’s duties can be sources of both negative and positive work-related experiences. Employees associate these experiences with various emotions, leading to differing moods that influence attitudes, behaviors, and work activities (Jaworek et al., 2020; Mishra & Venkatesan, 2023).

Some research has convincingly demonstrated that feelings play important roles in working conditions, organizational behavior, and leadership (Ilies et al., 2024). Social and sociocultural theories posit that feelings are not merely processes in the mind; they also shape and organize social interactions and their effects from socially oriented viewpoints (Schoeneborn et al., 2019). Feelings can be interpreted as social structures with contextual labels that relate to definitions shaped by culture, tradition, and daily experiences (Çakıt et al., 2020). People also view feelings as dynamic mechanisms that influence social activities, experiences, and their consequences, particularly in workplace contexts (Bisbey & Salas, 2019).

From organizational, psychological, and sociological perspectives, researchers have examined feelings at work, exploring how emotions influence behavior, job performance, and interpersonal relationships in the workplace. This field addresses the principles of emotional intelligence, emotional labor, and how employees manage and express their feelings in professional settings (Troth et al., 2018). Emotional intelligence refers to the degree to which one can manage one’s emotions and effectively direct one’s thoughts and actions. This process requires the ability to recognize and understand others’ feelings, as well as the capacity to use this knowledge to inform decision-making and behavior (Sanchez-Gomez & Breso, 2020).

The study of emotions at work encompasses two main aspects: (a) the use of emotional displays in individuals’ work roles to influence others towards achieving organizational goals, such as enhancing customer satisfaction, and (b) the generation of emotions that are acceptable to the organization (Çakıt et al., 2020). These aspects are closely linked to the identification of primary emotions, such as happiness, fear, anger, and sadness, which researchers have identified as fundamental to emotional experiences in the workplace (Hartmann et al., 2023). The intricate psychological makeup of these emotions is also associated with professional life and influences behavior within organizational settings (Zheng & Montargot, 2022). For instance, depressive symptoms and disorders, often linked to feelings of sadness, are associated with occupational burnout syndrome. Professional experiences profoundly intertwine with these basic emotions, with their intricate psychological framework (Yu et al., 2021). Employees who display symptoms of burnout may also experience anger, hostility, and aggressive behaviors (Ahola et al., 2014; Malik & Pichler, 2023). Studies show that work-related stress is one of the most serious problems that employees face today (Ul Hassan et al., 2023). At present, researchers possess access to only a limited array of validated instruments for examining emotional states within organizational contexts. These tools only address the negative and positive effects of emotions, ignoring the fact that each emotion can cause different tendencies and actions. Studying emotional reactions requires adequate measurement tools. Organizational settings view happiness as part of work-related well-being, which positively impacts employee job satisfaction, work participation, and organizational commitment (Luqman et al., 2023).

Researchers have developed various tools to measure people’s emotions in the workplace, allowing for an exploration of how these emotions manifest in a professional context (Lönn et al., 2023). The most common methods for assessing emotional states include pencil-and-paper tools (Eckford & Barnett, 2016) such as the Job-related Affective Well-being Scale (JAWS) (Van Katwyk et al., 2000), and comparisons between paper-and-pencil and internet survey methods conducted in combat-deployed environments, including the Job Affective Scale (Burke et al., 1993). These scales generally focus on either positive or negative emotions. While instruments like the JAWS and the Job Affective Scale provide valuable insights into general emotional states, they are insufficient for fully capturing the complexity of emotions in the workplace (Chen et al., 2024). These tools primarily assess overall affective well-being and job satisfaction, but they do not adequately account for specific emotions, such as fear and anger, which are linked to distinct behavioral patterns and organizational outcomes. Therefore, to comprehensively evaluate emotional dynamics at work, it is crucial to use a broader range of instruments that measure both general and specific emotions, along with their psychological and organizational implications.

In 2020, Jaworek and colleagues developed the Work-Related Affective Feelings (WORAF) Scale to measure emotional states in organizational settings. The concept of Work-Related Affective Feelings refers to the emotional experiences directly linked to one’s occupational activities, roles, and environments. These affective states encompass primary emotions such as happiness, anxiety (fear), anger, and dejection (sadness), which are frequently elicited in workplace contexts. Historically, the study of emotions in organizations was initially centered on broad constructs like job satisfaction and general well-being (Burke et al., 1993; Van Katwyk et al., 2000). Early instruments often categorized emotions into positive and negative affects without accounting for the specific emotional nuances associated with distinct work situations. Over time, organizational psychologists recognized that discrete emotions have unique antecedents, behavioral consequences, and organizational implications (Ashkanasy & Kay, 2023). The development of the WORAF Scale addressed this need by uniquely measuring four primary emotional states rather than general affective categories. WORAF’s conceptualization marks an important advancement by emphasizing the role of fundamental emotions in understanding employee experiences, moving beyond the binary positive–negative framework toward a more differentiated emotional analysis in occupational settings. The WORAF Scale distinguishes itself from other scales by focusing on primary emotions rather than solely categorizing them as positive or negative. It measures feelings of happiness, anxiety (fear), anger, and dejection (sadness) (Ashkanasy & Kay, 2023).

Although the WORAF Scale demonstrates strong psychometric properties (Jaworek et al., 2020), it remains essential to validate and adapt this tool within the Iranian cultural context. Despite growing global recognition of the importance of emotional experiences at work, a significant research gap persists in Iranian organizations. Previous studies have predominantly focused on broad constructs like job satisfaction, organizational commitment, and occupational stress, while often overlooking discrete emotional experiences. There is also a critical shortage of validated, culturally sensitive tools for assessing specific emotions, such as happiness, fear, anger, and sadness in Iranian workplaces.

Given the unique sociocultural characteristics of Iranian organizations, such as hierarchical values, a focus on social harmony, and emotional restraint, the need for culturally appropriate measurement tools is crucial. Emotions like fear and anger may have distinct implications for employee behavior, interpersonal relationships, and leadership dynamics in Iranian workplaces. For instance, in environments where respect for hierarchy and social order is prioritized, emotions such as anger and anxiety could significantly impact team dynamics, communication, and overall organizational outcomes.

The Farsi version of the WORAF Scale, by offering a nuanced and reliable method of assessing emotions, addresses this gap. It enhances the understanding of emotional dynamics in Iranian workplaces, contributing to evidence-based management practices aimed at improving employee well-being and organizational effectiveness. By measuring emotions like happiness, fear, anger, and sadness, organizations can develop more targeted strategies to address emotional challenges, reduce stress, and create a supportive work environment. This can improve job satisfaction, organizational commitment, and productivity.

Therefore, this study aims to examine the psychometric properties, validity, and reliability of the Farsi version of the WORAF Scale within Iranian organizations. This research provides a culturally relevant tool for assessing emotional dynamics and offers practical insights for enhancing employee well-being and improving organizational outcomes in the Iranian workplace.

## Methods

This research employed a descriptive-correlational method.

### Participants

This study involved 514 participants, who were employed men and women across various industries in Iran, recruited using convenience sampling from January to July 2023.

Participants were selected from a broad range of organizations, including but not limited to private companies, public sector organizations, educational institutions, government agencies, healthcare and medical institutions, and non-governmental organizations (NGOs). The sample consisted of both employees and managers, with a balanced representation of workers across different roles within these organizations. In terms of demographic characteristics, the sample was almost equally divided between men and women: 48.6% (*n* = 250) of participants were female and 51.4% (*n* = 264) were male. Regarding their employment type, 62.3% (*n* = 320) of participants were full-time employees, while 37.7% (*n* = 194) were employed part-time. The sample included employees from various job positions, including both non-managerial and managerial roles. While majority were regular employees (*n* = 420), a significant portion (*n* = 94) held managerial or leadership positions. Regarding work experience, participants were categorized as follows: 29.6% (*n* = 152) had less than five years of work experience, 54.5% (*n* = 280) had between five and ten years of experience, and 15.9% (*n* = 82) had over ten years of work experience. The participants’ education levels varied, with 24.5% (*n* = 126) having completed high school but not graduated, 10.0% (*n* = 51) holding associate’s degrees, 43.3% (*n* = 223) holding bachelor’s degrees, and 22.2% (*n* = 114) holding master’s degrees or higher. All participants were employed either full-time or part-time during the study and were required to have been employed for at least six months to qualify for participation.

### Procedure

The survey was administered electronically to the 514 participants across various industries in Iran from January to July 2023. A dual recruitment strategy was employed, combining online channels (e.g., industry-specific social media groups, LinkedIn^*^, Instagram^*^, Telegram, WhatsApp, and SMS) and organizational partnerships. This was distributed via a secure Google Forms platform, with participants accessing the questionnaire through links shared across these recruitment channels. These platforms ensured broad representation across sectors and geographic regions, while organizational channels targeted employees in private companies, government agencies, educational institutions, healthcare, and NGOs. The inclusion criteria required participants to be employed full-time or part-time in any industry or sector in Iran, have worked for at least six months during the study period, and be fluent in Farsi. Participants also needed to provide written informed consent.

Before the main study, the WORAF Scale underwent a rigorous translation process using Brislin’s back-translation method (Brislin, 1980; Jones et al., 2001). Two bilingual experts, one specializing in psychology and the other in English language studies, conducted the translation and back-translation process. The translated Farsi version was reviewed for face validity by university professors and psychology experts, and any discrepancies were addressed to ensure linguistic accuracy and conceptual clarity. A pilot study involving 25 participants was conducted to assess the clarity, cultural appropriateness, and comprehensibility of the translated items (Cronbach’s alpha ≥ .78). Feedback from these participants led to refinements in the survey. Based on this feedback, minor modifications were made to improve item clarity and reduce survey completion time to 15 minutes.

Once the survey was finalized, all questions were made mandatory, preventing the submission of incomplete forms and minimizing missing data. Participants were allowed to review and modify their responses before final submission to ensure accuracy. To maintain data quality, participants were given 14 days to complete the survey, with reminders sent every four days to non-respondents. After data collection, responses were screened for inconsistencies and outliers using SPSS-26, and invalid responses were removed. Confirmatory Factor Analysis (CFA) and reliability were conducted to ensure measurement accuracy. No personally identifiable information was collected, and all responses were encrypted and stored securely, ensuring confidentiality. This comprehensive approach, including pre-testing, pilot validation, and rigorous data cleaning, ensured data quality and minimized potential biases for robust descriptive-correlational analysis.

### Questionnaires

#### The Work-Related Affective Feelings (WORAF) Scale

Jaworek et al. (2020) developed this scale to measure emotional states, consisting of 24 questions. WORAF uses a Likert point scale ranging from totally agree (5) to totally disagree (1) and looks into the four main feelings of anxiety (fear), happiness, dejection (sadness), and anger (Jaworek et al., 2020). The main study by Jaworek et al. (2019) demonstrated proper scale validity. Also, the researchers showed excellent reliability values using Cronbach’s alpha for each factor (subscales), ranging from .78 for sadness to .83 for anxiety (Jaworek et al., 2020).

#### The Job-related Affective Well-being Scale (JAWS)

Van Katwyk et al. (2000) developed the Job-related Affective Well-being Scale (JAWS) to measure positive and negative emotions in response to various aspects of their jobs, including the job itself, colleagues, boss, clients, and salary. There are two versions of JAWS. The long version contains 30 items, and the short one has 20 items. This study used the 20-item form. JAWS had a 5-item Likert point scale ranging from “never” to “always.” A study estimated the Cronbach’s alpha for JAWS to range between .80 and .95 (Van Katwyk et al., 2000). An analysis of the items in JAWS revealed that the scale’s reliability with Cronbach’s alpha and halving methods was .92 and .91 for positive emotion, and .93 and .92 for negative emotion. This indicates that JAWS has optimal reliability (Babamiri et al., 2021). Another study obtained validity coefficients of .91 for this questionnaire using internal consistency and .90 using the Guttman split-half method (Donaldson & Donaldson, 2020). In 2022, a study calculated the correlation coefficients of this scale with interpersonal conflict and job satisfaction and found them to be -.32 and .81 (Falatah & Alhalal, 2022). In the present study, the reliability of scale was .76 using Cronbach’s alpha.

#### Ten-Item Personality Inventory (TIPI)

Gosling et al. (2003) developed the Ten-Item Personality Inventory (TIPI) to assess individuals’ personality traits. The scale comprises 10 items that measure five major dimensions of personality: extraversion, agreeableness, conscientiousness, emotional stability, and openness to experience. Each dimension is assessed using two items, with responses rated on a 7-point Likert scale ranging from 1 (strongly disagree) to 7 (strongly agree) (Gosling et al., 2003). Some studies (Rostami et al., 2022; Thørrisen & Sadeghi, 2023) have shown the reliability was .76 using Cronbach’s alpha. In the present study, reliability of the TIPI was found to be .74 using Cronbach’s alpha.

### Data Analysis

To assess the psychometric quality of the WORAF scale, we adopted the classical approach of test analysis focusing on validity and reliability. Prior to performing statistical analyses, we investigated the assumptions of multivariate normality and the absence of multivariate outliers. To analyze the data, we calculated the skewness and kurtosis indices to assess normality. The variables’ skewness varied from -.4 to 1.30, and their kurtosis varied from -.66 to 1.33. A skewness cut-off point of ±3 is considered appropriate, while values exceeding ±10 for kurtosis are generally regarded as problematic (Chou & Bentler, 2002). We established normality based on the obtained values for skewness and kurtosis. We used SPSS software version 26 for descriptive and demographic information analysis and R software version 4.2 (semPlot and lavaan packages) for the CFA. We analyzed the data using CFA, Pearson correlation coefficient, and Cronbach’s alpha.

## Results

To investigate the face validity of WORAF, the research used both qualitative and quantitative methods. A five-member panel, comprising two psychologists and three university professors, assessed the qualitative face validity. The panel evaluated aspects such as the degree of difficulty, disproportion, ambiguity in phrases, and semantic anomalies in words. Based on the expert opinions, we made minor modifications to the scale. To determine the quantitative face validity of each question, we calculated its impact score. The 24 questions were rated on a five-point Likert scale (0 = “I fully disagree” to 5 = “I fully agree”). We then administered the scale to 10 individuals to evaluate its validity. After the target group completed the scale, we calculated the face validity using the impact score formula, and confirmed that all questions were proportionate in terms of face validity. To assess the content validity, we asked five experts to review the scale’s items and provide written feedback. They assessed factors such as grammatical accuracy, proper vocabulary usage, the significance of each question, appropriate question placement, and the time required to complete the scale. After receiving the experts’ feedback, we made the necessary revisions to the scale.

We used two content validity ratios (CVRs) and a content validity index (CVI) to evaluate the content validity (Wynd et al., 2003). In order to evaluate the CVR, we asked the expert group to evaluate each question using a three-component spectrum: “necessary, useful, and unnecessary.” Finally, using Equation 1, we calculated the CVR based on the answers.


$$\text{Equation 1-CVR}=\frac{n_{e}-\frac{N}{2}}{\frac{N}{2}},$$

*n_e_* — Number of specialists who have selected the necessary option: *N* — Total number of specialists.

The number of specialists also influences the acceptable range; in this study, with 10 specialists, we set the threshold at .62 (DeVon et al., 2007). In other words, we confirm the content validity of a question if its calculated CVR equals or exceeds .62. The present study calculated the CVI using the mean CVR of all remaining questions, as shown in Equation 2 (Lawshe, 1975). If the CVI value exceeds .79, it confirms the validity of the scale content.


$$\text{Equation 2-CVI}=\frac{\sum \text{CVR}\;\text{for}\;\text{all}\;\text{retained}\;\text{items}}{\text{retained}\;\text{items}\;number}.$$

Expert opinions in this study indicate that, out of the 24 questions in the WORAF, 23 had a CVR higher than .62, with the exception of question 15. We calculated the CVI of the remaining 23 questions to be .93. Since the CVI threshold for question acceptance is .79, we consider this value acceptable for the overall scale.

The CVR analysis of the WORAF Scale produced compelling results. Out of 24 items, 23 exceeded the critical CVR threshold of .62, demonstrating strong content validity for most of the scale. However, item 15 fell below this threshold. The mean CVR of the remaining 23 items calculated the CVI, which was .93, well above the .79 acceptance criterion. This high CVI score underscores the overall content validity of the scale. Descriptive statistics provided further insight into the scale’s properties. Mean scores ranged from 1.61 (item 18: “Most work-related activities make me feel sad and useless”) to 2.73 (item 10: “My job brings me satisfaction”), capturing a broad range of affective experiences. Standard deviations between .77 and .98 indicate appropriate response variability. Items related to job satisfaction (items 9–11) and work-related anxiety (items 1–4) showed relatively high mean scores, underscoring their importance in the overall construct of work-related affect. The scale balances positive and negative affect items, as seen in contrasting statements like “I find my work enjoyable” (item 9, mean = 2.61) and “At work, I feel like I have reached the bottom” (item 16, mean = 1.68). Lower means for negative affect items (e.g., items 16–18 and 22–24) suggest that participants, on average, experienced negative work-related feelings less frequently or intensely than positive ones.

**Figure 1:**
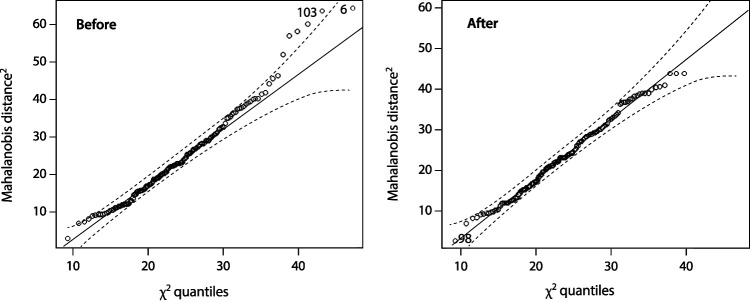
Mahalanobis distance index to check normality and the absence of outlier data

To assess the presence of outliers, a Quantile-Quantile (Q-Q) plot was utilized. We further supported this analysis by calculating the Mahalanobis distance for each data point. *Figure 1* visualizes these analyses.

In *Figure 1*, the presence of multiple multivariate outputs hindered the establishment of normality. After removing this data, we successfully established multivariate normal conditions.

To evaluate the validity of the scale, we employed four methods of construct validity: criterion validity, convergent validity, and discriminant validity. We further investigated the construct validity of WORAF using the CFA method.

We measured the construct validity of WORAF using the maximum likelihood method in CFA. We assessed the factor loadings of the items and eliminated any with factor loading below .40 (Martynova et al., 2018). *Table 1* demonstrates that no WORAF items were excluded. The goodness-of-fit indices were as follows: CFI (Comparative Fit Index) = .907, TLI (Tucker-Lewis Index) = .904, IFI (Incremental Fit Index) = .907, X^2^/df = 3.809, RMSEA (Root Mean Square Error of Approximation) = .079, 95% CI = (.074–.083), and SRMR (Standardized Root Mean Squared Residual) = .055. These indices confirmed that the factor structure of WORAF fits the data well. *Table 1* also reports the standardized factor loadings, z-statistics, and significance levels for each item.

**Table 1:** Factor Loading, Z-Statistics, and Significance of WORAF’s Items

Factor	Item	factor load	Z statistic	*p*
Anxiety	1	.774	–	–
	2	.546	11.22	.001
	3	.721	15.38	.001
	4	.812	17.74	.001
	5	.727	15.53	.001
	6	.726	15.5	.001
	7	.799	17.41	.001
	8	.74	15.86	.001
Happiness	9	.805	–	–
	10	.847	20.08	.001
	11	.868	20.77	.001
	12	.77	17.61	.001
	13	.877	21.09	.001
	14	.708	15.76	.001
	15	.435	8.92	
Dejection	16	.79	–	–
	17	.87	19.76	.001
	8	.664	14.12	.001
	19	.728	15.78	.001
	20	.857	19.38	.001
Anger	21	.66	–	–
	22	.899	15.62	.001
	23	.837	14.81	.001
	24	.889	15.5	.001

**Figure 2:**
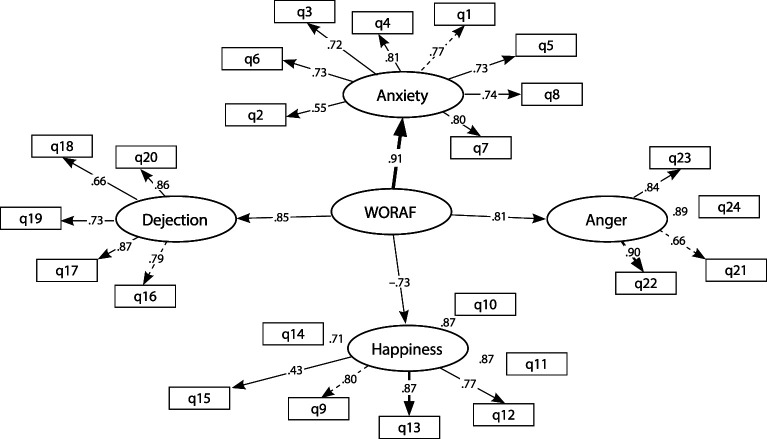
The Confirmatory Factor Analysis (CFA) model of WORAF

As shown in *Table 1*, the z-statistics for all items are significant at the .05 level. *Figure 2* presents the validated CFA model.

To assess the criterion validity of WORAF’s dimensions, we calculated the correlation between the scores of JAWS and TIPI.

*Table 2* presents correlations between dimensions of WORAF, TIPI, and JAWS. The strongest and weakest significant correlation coefficients between dimensions of WORAF and TIPI were observed for anxiety and extraversion (*r* = –.313) and conscientiousness and anxiety (*r* = -.205). No significant relationships were found between any of dimensions of WORAF Scale and JAWS. The strongest correlations were between positive feelings and positive emotion (*r* = .819) and anxiety and negative emotion (*r* =.741), indicating strong associations between WORAF’s dimensions and positive and negative emotions.

**Table 2:** Correlation between Dimensions of WORAF and TIPI and JAWS

		WORAF components			
		Anxiety	Happiness	Dejection	Anger
Dimensions of TIPI	Extraversion	–.313^**^	.306^**^	–.274^**^	–.275^**^
	Agreeableness	–.304^**^	.162^**^	–.199^**^	–.26^**^
	Conscientiousness	–.205^**^	.134^**^	–.177^**^	–.137^**^
	Emotional stability	–.144^**^	.161^**^	–.122^*^	–.122^*^
	Openness to experience	–.243^**^	.43^**^	–.236^**^	–.277^**^
JAWS	Positive emotion	–.468^**^	.82^**^	–.48^**^	–.49^**^
	Negative emotion	.741^**^	–.697^**^	.671^**^	.554^**^

Note. **Correlation is significant at the .01 level (2-tailed)

### Convergent and Discriminant Validity

We used Average Variance Extracted (AVE) to evaluate the convergent validity, with value greater than .5 indicating adequacy. The square root of the AVE for each latent variable is located on the diagonal of the correlation matrix, while the off -diagonal cells represent the correlations between variables. If the AVE for each latent variable (on the diagonal) exceeds the squared correlation between that variable and other variables, it confirms discriminant validity.

As shown in *Table 3*, the AVE values for all dimensions of WORAF are above the recommended threshold of .5, confirming convergent validity. The square roots of the AVE (diagonal values in the matrix) are higher than the other values, indicating that the latent variables in the research model are more strongly associated with their respective items than with other constructs, which supports the model’s discriminant validity.

**Table 3:** Results of WORAF’s Convergent and Discriminant Validity

Variables	AVE	Anxiety	Happiness	Dejection	Anger
Anxiety	.53	.72			
Happiness	.59	–.59	.76		
Dejection	.61	.68	–.58	.78	
Anger	.68	.66	–.55	.62	.82
Total score	.60				

We assessed the reliability and internal consistency of WORAF using composite reliability, Cronbach’s alpha coefficient, and test-retest reliability. Both composite reliability and Cronbach’s alpha should exceed .70 to demonstrate adequate reliability, and all values met this threshold, confirming the scale’s reliability.

The data analysis indicates that the WORAF Scale and its dimensions demonstrated high composite reliability and internal consistency *(Table 4)*. To evaluate the test-retest reliability of the WORAF, a follow-up study was conducted with 159 participants (mean age = 35.91, SD = 5.46). Intra-Class Correlation Coefficients (ICCs) were calculated for each item and the total score. ICCs for individual items ranged from .68 to .913, reflecting good to excellent reliability across all items. Specifically, 21 out of 24 items had ICCs above .70, with 11 items showing ICCs above .80, suggesting strong stability at the item level. The lowest ICC was observed for item 21 (ICC = .68), and the highest ICC was for item 18 (ICC = .913).

**Table 4:** Reliability of Data Using Combined Reliability and Cronbach’s Alpha

Variables	CA	CR
Anxiety	.92	.73
Happiness	.89	.84
Dejection	.92	.85
Anger	.88	.83
WORAF	.87	.80

*Note. CA: Cronbach’s alpha, CR: Composite Reliability*

Mean scores for individual items in the initial test ranged from 1.61 to 2.71, with standard deviations between .742 and 1.09, while in the retest, mean scores ranged from 1.56 to 2.65, with standard deviations between .725 and 1.007. The total score across both test and retest demonstrated an ICC of .84, indicating excellent overall scale reliability. These findings provide robust evidence of the temporal stability of the WORAF Scale, supporting its use in longitudinal studies and repeated measures designs.

## Discussion

The present study aimed to determine psychometric properties of the Farsi version of the Work-Related Affective Feelings (WORAF) Scale in the Iranian sample. This research demonstrated that the Farsi version of the WORAF Scale had acceptable validity and reliability. We conducted a CFA to validate the factor structure of the scale. The factor loadings showed that the WORAF Scale is appropriate and fits well with the measurement model (Jaworek et al., 2020).

It is worth noting that item 15 was removed during the content validity step due to its CVR being below the acceptable threshold. This finding differs from the original version, in which item 15 was retained. The removal in the current study reflects cultural or linguistic differences that may have affected the perceived relevance or clarity of this item in the Iranian context.

Our findings revealed a significant relationship between the dimensions of the WORAF Scale, TIPI, and JAWS. The inclusion of the TIPI (Ten-Item Personality Inventory) and JAWS (Job-related Affective Well-being Scale) alongside the WORAF Scale was intentional and theoretically grounded. TIPI was selected to examine the relationship between personality traits and affective experiences at work, based on extensive literature suggesting that traits such as neuroticism and extraversion significantly influence emotional responses in organizational contexts. On the other hand, JAWS was included as it is a well-established measure of job-related emotions, providing a useful benchmark for evaluating the convergent and discriminant validity of the WORAF Scale. Comparing WORAF with these instruments allowed us to assess whether the newly adapted Farsi version not only captured unique dimensions of workplace affect but also aligned with existing constructs in personality and affective science.

This triangulation strengthens the construct validity of WORAF and enhances our understanding of emotional functioning in the workplace within a sociocultural context. This suggests that the emotional states assessed by the WORAF Scale are not only reliable, but also relevant to understanding workplace dynamics. The positive correlations observed imply that individuals who experience positive work-related feelings may also exhibit favorable personality traits and greater job-related well- being. The strongest and weakest significant correlation coefficient regarding the relationship between the dimensions of WORAF Scale was related to anxiety and extroversion and conscientiousness and anxiety. All dimensions of the WORAF Scale have a significant correlation with JAWS. The strongest correlation coefficients between the WORAF’s dimensions and negative and positive emotions are between positive feeling and positive emotion and anxiety and negative emotion.

These findings are in line with previous research, which has further supported the correlations observed in this study. For example, experimental studies have consistently demonstrated similar patterns between emotional states in the workplace and personality traits, validating the relationships identified in our study (Basinska et al., 2014; Jaworek et al., 2020). These studies highlight the significant role of work-related emotions in shaping organizational behavior and employee well-being. In particular, Jaworek et al. (2020) found strong correlations between positive emotional states and job satisfaction, highlighting the significance of tools like the WORAF Scale in capturing these dynamics.

These findings can also be interpreted through the lens of social and sociocultural theories, which emphasize that emotions are not merely internal states, but are socially constructed and shaped by cultural norms, traditions, and workplace environments. In the Iranian context, where social harmony, emotional restraint, and hierarchical structures are culturally embedded, emotions like anger or anxiety may manifest differently compared to Western settings. The strong psychometric performance of the WORAF Scale in this study suggests that, although core emotions like happiness, fear, anger, and sadness are universal, their expression and organizational consequences are culturally nuanced. For instance, the significant relationship between anxiety and negative emotions may reflect culturally influenced emotional suppression or fear of conflict in hierarchical settings. Thus, our findings support the idea that emotional experiences at work are psychologically valid and socially shaped, reinforcing the value of sociocultural theories in interpreting work-related affective dynamics.

By corroborating these findings, our research adds to the growing body of evidence that emphasizes the predictive value of emotional assessments in understanding workplace outcomes. The WORAF Scale, a multi-item measurement tool, is a new instrument that meets the expectations of other researchers by measuring core yet work-related emotions (Alsughayir, 2021). Different emotional states can lead to distinct organizational behaviors and consequences. Researchers interested in expanding knowledge about the psychological functioning of individuals in the workplace, as well as those interested in studying each of the core emotions in the workplace, can use the WORAF Scale (Jaworek et al., 2020).

These results have implications that go beyond mere measurement. The WORAF Scale’s reliability in a Farsi context enhances its applicability for practitioners and researchers aiming to assess emotional experiences in the workplace. Understanding the interplay between personality traits and affective experiences can inform interventions aimed at improving workplace culture and employee engagement. Future research could explore how specific affective states impact job performance and overall employee satisfaction. By leveraging the insights from the WORAF Scale, organizations can better tailor their approaches to fostering positive emotional experiences among employees.

## Conclusion

The findings suggested that the WORAF Scale has an acceptable level of validity and reliability in Iran. This means that this scale can provide researchers with a reliable and valid method for measuring various emotions experienced by individuals in the workplace. In conclusion, the WORAF Scale is a suitable tool for researchers and human resource units to gain original knowledge and insights into the emotions employees experience at the workplace, intending to promote positive emotions and reduce negative emotions in the workplace. It should be noted that this scale has not been previously validated or used in Iran, pointing to the originality of this study and providing a standard research scale for future joint research between Iran and other countries.

## Implications

This scale can enhance our understanding of the relationship between emotions and job performance, job satisfaction, and overall well-being by assessing feelings such as joy, boredom, anxiety, and anger, facilitating more effective research while minimizing participant burden, which can be crucial for informing mental health initiatives and human resource strategies. For example, organizations can use the insights gained from WORAF to identify employees who may be at risk of burnout, anxiety, or dissatisfaction, allowing for timely interventions such as stress management programs, counseling services, or team-building exercises to promote positive emotional experiences at work. Moreover, the scale’s ability to assess positive and negative emotions can guide the development of policies that foster more supportive and emotionally healthy workplace environment. Human resources departments could integrate the findings into training programs that enhance emotional intelligence, improve communication, and reduce workplace conflicts. By doing so, the organization can improve job satisfaction, employee retention, and overall productivity. In a broader context, the use of WORAF in various industries could contribute to national efforts to improve workplace well-being, potentially influencing government policies on occupational health and safety. Thus, the scale provides not only academic insights but also practical tools for fostering healthier, more emotionally supportive workplaces.

## Limitations

One of the key limitations of this study is the lack of including all available jobs in Iran, which affects the generalizability of the results. This study employed convenience sampling, a non-probability sampling method where participants are selected based on availability and accessibility (Andrade, 2021), it is important to acknowledge that this approach has both advantages and limitations. While convenience sampling offers practical benefits such as cost-effectiveness and efficiency in data collection, it may introduce sampling bias, as it does not ensure a representative sample of the broader population (Campbell et al., 2020). The findings of this study may have limited generalizability beyond the study sample. However, the diversity of participants across various industries, organizational types, and employment conditions in this study provides valuable insights into the emotional well-being of employees in these contexts. Future research utilizing probability sampling methods may help confirm and extend these findings to a broader population.

While the sample included participants from the private sector, government agencies, businesses, and small companies, it may not fully represent the diverse experiences of employees working in industries such as healthcare, education, manufacturing, or technology. For instance, employees in high-stress environments like hospitals or schools might experience different emotional states at work compared to those in office-based roles or less demanding industries. Although this study did not specifically address it, the emotional demands of jobs that involve direct interaction with clients or patients, like nursing or customer service, could significantly influence the results. Future research is suggested to include a more diverse representation of job types to ensure a broader understanding of how work-related affective feelings vary across various sectors and occupational environments in Iran.

Although using online data collection platforms is widely accepted in psychological research, it may bring with it response biases, particularly social desirability bias, where participants might answer questions in a way that aligns with social norms or expectations. In studies related to the workplace, employees may express concerns that disclosing negative emotions or dissatisfaction could jeopardize their job security, despite the assurance of anonymity. Online surveys may also limit participation to individuals with access to technology and the internet, excluding those who are less tech-savvy or who do not have regular internet access. This could lead to a sample that is not fully representative of the broader working population; particularly in less urbanized or lower-income regions. The online format can result in misunderstandings of the survey questions, as there is no facilitator present to clarify ambiguities, which might affect the accuracy of the responses. Therefore, future studies should consider complementing online data collection with other methods, such as face-to-face interviews or paper-based surveys, to ensure a more inclusive and reliable data set.

In addition, when asking people to complete questionnaires about their workplace emotions, there is always the question of how reliably they can describe these experiences. Despite the extensive use of self-report methods in personality research and individual differences, it is crucial to employ multiple strategies when studying workplace emotions. Future studies should include the WORAF Scale alongside other tools such as those measuring cognitive tasks (Joormann & Quinn, 2014).

## Footnotes

* Activities of Meta Platforms Inc. (Facebook and Instagram), the X Corp. (as the successor of Twitter Inc.), and LinkedIn are prohibited in the Russian Federation.
